# Developmental performance of hospitalized severely acutely malnourished under-six children in low- income setting

**DOI:** 10.1186/s12887-017-0950-5

**Published:** 2017-11-28

**Authors:** Teklu Gemechu Abessa, Liesbeth Bruckers, Patrick Kolsteren, Marita Granitzer

**Affiliations:** 1 Department of Special Needs and Inclusive Education, College of Behavioral Sciences and Education, Jimma University, Jimma, Ethiopia; 2 I-Biostat, Hasselt University, Hasselt, Limburg Belgium; 3 Department of Food Safety and Food Quality, University of Gent, Ghent, Belgium; 4 REVAL Rehabilitation Research Center, Biomedical Research Institute, Faculty of Medicine and Life Sciences, University of Hasselt, Hasselt, Belgium

**Keywords:** Developmental performance, Child development, Severe acute malnutrition

## Abstract

**Background:**

Retrospective studies show that severe acute malnutrition (SAM) affects child development. However, to what extent SAM affects children of different ages at its acute stage is not well documented. This study was aimed at comparing the developmental performance of severely acutely malnourished children under six with that of age and gender-matched non-malnourished healthy children.

**Methods:**

The developmental performances of 310 children with SAM (male = 155, female = 155); mean age = 30.7 mo; SD = 15.2 mo) admitted to the nutritional rehabilitation unit (NRU) at Jimma University’s Hospital was compared with that of 310 age and gender-matched, non-malnourished healthy children (male = 155, female = 155; mean age = 29.6 mo; SD = 15.4 mo) living in Jimma Town in Ethiopia. Two culturally adapted tools were used: (1) the Denver II-Jimma, to assess the children’s performance on personal social (PS), fine motor (FM) language (LA), gross motor (GM) skills, and (2) the Ages and Stages Questionnaires: Social-Emotional (ASQ:SE), to assess social-emotional (SE) skills. Multivariable Poisson regression analysis was conducted to compare the developmental performance scores of SAM and non-malnourished children.

**Results:**

For one-year-old children, SAM delays their developmental performance on GM, FM, PS and LA by 300%, 200%, 140% and 71.4% respectively. For three-years-old children, SAM delays their developmental performance on GM by 80%, on FM and LA by 50% each, and on PS by 28.6%. Of the skills assessed on Denver II-Jimma, GM is the most, and PS is the least affected. Younger SAM children are more affected than older ones on all the domains of development. The delay in FM, GM, LA and PS generally decreases with an increase in age. Social-emotional behavior problems seem to be most pronounced in the very young and older age ranges.

**Conclusions:**

SAM has a differential age effect on the different dimensions of development in children under 6 years of age.

**Electronic supplementary material:**

The online version of this article (10.1186/s12887-017-0950-5) contains supplementary material, which is available to authorized users.

## Background

Child undernutrition, which manifests mainly as stunting, underweight, and wasting is one of the global health problems. In 2013, at least 161 million under-five children were globally stunted; 99 million were underweight; and 51 million were wasted with a higher prevalence in Asia and sub-Saharan Africa [1]. In Ethiopia, a report in 2014 shows that 40%, 25% and 9% of under-five children were stunted, underweight and wasted respectively [2]. Though under-five, infant and neonatal mortality has declined from 205 deaths in 1990 to only 64 deaths per 1000 live births in 2013 [3], Ethiopia is still among the 14 countries in the world with the largest burden and highest prevalence of stunting, and among 10 countries with the highest prevalence of wasting [4].

Studies have documented the detrimental effects of child malnutrition on growth, development, later school achievement, and health outcomes [5–14]. Most of such studies focused on chronic malnutrition. Some studies addressed long-term effects of severe malnutrition using a retrospective case control study design [15–21] and focused only on one or very few dimensions of development. Few studies that dealt with the short-term effects of severe acute malnutrition [22–24] compared hospitalized severely malnourished and non-malnourished control subjects or siblings. The levels of the different dimensions of development of such children have not been investigated by comparing them with that of healthy children developing in optimal conditions. Moreover, since indigenous tools for assessing child development are lacking in low-income contexts such as countries in Africa, research in the area of child development is very scarce. The very few studies conducted have used Western tools without standardizing them on local children by either translating or adapting them with little validation [25–29], or by dropping culture specific test items [5, 6, 30–32]. The validity and reliability of data obtained using such tools is thus questionable. With the idea that the first 3 years of life is the critical period for child development, many studies focused on infants and young children of three or less years of age. Moreover, such studies have not addressed various developmental dimensions simultaneously. Hence, studies comprehensively addressing multiple dimensions of child development by including children above 3 years of age are scarce. Consequently, it is unclear whether or not risk factors such as severe acute malnutrition differentially affect the different dimensions of development in children under six.

In brief, in low- income settings, there is a lack of more reliable and multidimensional insight into the comprehensive developmental profile of under-six children at risk such as those with severe acute malnutrition (SAM).

The aim of this study, therefore, was to compare multidimensional developmental performance of 3 months to 6 year old severe acute malnourished (SAM) children with non-malnourished healthy children using culturally adapted tools.

## Method

### Study setting, design and sampling

The study was conducted in Jimma Zone, south west Ethiopia. According to the 2007 census [33], Jimma Zone has 17 districts having a population of 2,486,155 (50.3% male). Majority (94.5%) live in rural areas on subsistence agriculture; 2,129,321 (85.6%) are followers of Islam. The zonal capital, Jimma Town, has a population of 120,960 (50.3% male). The majority (56,661 or 46.8%) of residents of Jimma Town are Orthodox Christians; 47,205, or 39% are Muslims, and 15,799 or 13.1% are protestant Christians. Cross-sectional data were collected from both severely acutely malnourished (SAM) and non-malnourished healthy children. SAM children admitted to hospital for treatment were recruited with a non-probability convenient sampling. Age and gender matched non-malnourished healthy children were selected purposefully from families with middle or high socio-economic status assumed to be suitable for optimal child development. The SAM and the non-malnourished groups were assessed using culturally adapted tools and compared on five different areas of child development.

### Participants

#### Severe acute malnourished (SAM) children

**Fig. 1:**
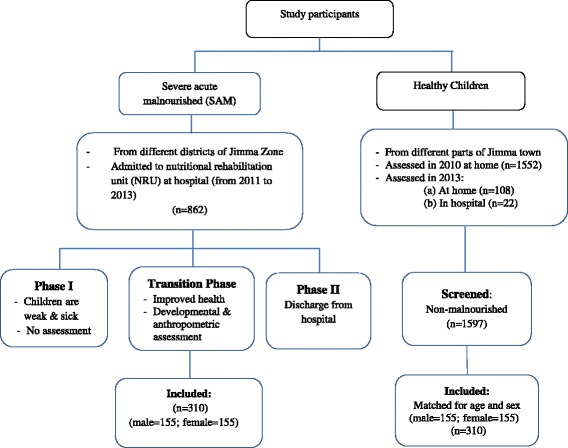
Selection of study participants

A total of 826 SAM children were coming from nearby districts in Jimma Zone and admitted to the nutritional rehabilitation unit (NRU) at the pediatric ward of Jimma University’s Specialized Referral Teaching Hospital from 8/02/2011 to 28/04/2013. Only 310 (155 male, 155 female) children (mean age = 30.7 mo; SD = 15.2 mo; range = 3.1—65.7 mo) were involved in the study (see Fig. 1). Inclusion criteria were based primarily on a protocol prepared by the Ethiopian Federal Ministry of Health [34]: children (a) whose wasting was severe (weight-for-height [W/H] less than 70%, National Centre for Health Statistics (NCHS) [35]), or (b) with a low mid upper arm circumference (MUAC), i.e., MUAC less than 110 mm with a length greater than 65 cm; or, (c) having bilateral pitting edema. Only 3 months to 6 years of age children living within accessible driving and/or walking distance in the different districts of Jimma Zone were included. In case of twins, only one child was randomly chosen. Children with obvious disabilities, mobility problems and sensory impairments (hearing and visual problems) were excluded.

Of the three phases (stabilization, transition and rehabilitation) in the treatment of SAM children [34], developmental and anthropometric assessment were made during the transition phase. SAM patients cannot be tested during the first phase since they are without adequate appetite and /or have severe medical complications. Assessment was made when the patients had good appetite and no major medical complications.

### Non-malnourished (healthy) children

From a total of 1682 apparently healthy children under six who belong to families with middle or higher socio-economic status in Jimma Town, 310 children were selected and matched for age and gender with the severely malnourished children. Parental socio-economic status was determined using child’s access to preschool education as a proxy. The children of parents not affording payment for preschool education were excluded assuming that they belong to lower socio-economic status. A-10-point checklist was used to exclude the following potentially developmentally at-risk children: prematurely born, birth weight less than 2500 g, very tiny body at birth, instrumentally delivered, or delivered after 24 h of labor, born with chronic health problem, sick during the first year after birth, having observable impairments affecting sight or/and hearing, or/and mobility, having a mother who was seriously sick during pregnancy. In case of twins, one child was randomly excluded. Children suspected to be malnourished were excluded using weight-for-age and MUAC z-scores in line with WHO 2006 child growth standards [36].

#### Outcomes, measurements and instruments

Five areas of child development were looked into: fine motor (FM), gross motor (GM), language (LA), personal social (PS) and social-emotional (SE) skills***.*** The first four were assessed using the Denver II-Jimma [37]: a tool adapted to the Jimma context from the Denver II [38]. No test item was dropped during the adaptation. Test item administration and raw scoring is similar as in Denver II [39]. For each domain, the number of test items successfully performed by a child was counted.

The SE competences (self-regulation, adaptive functioning, affect, compliance, autonomy, interaction with people and communication behaviors) were assessed using parent completed Ages and Stages Questionnaire: Social-Emotional (ASQ:SE) [40] adapted to the study context (unpublished). For each item, a score equals zero if no problem is reported. A total score below an age specific cutoff indicates a typical behavior of a child, and above this cutoff indicates a presence of social-emotional problems.

Socio-demographic variables such as maternal education, socio-economic status, child sex and age were documented through a structured questionnaire because they were identified in earlier studies [41–44] as potential predictors of child developmental outcomes. Electronic digital weight scale and MUAC tape were used respectively to measure weight and MUAC of children.

### Data collection and testing procedure

Data were collected by five pairs of clinical nurses trained in anthropometric measurements and administrations of the ASQ:SE and Denver II-Jimma test items. The testing procedure was as follows: 1) interviewing parents or caregivers using a questionnaire on socio-demographic information, and the ASQ:SE; 2) testing the child with the Denver II-Jimma test; and, 3) finally, measuring weight and then MUAC.

### Statistical analysis

The primary goal was to investigate whether developmental performances of severely malnourished and non-malnourished children differ. The five developmental outcomes were summarized as count scores. Hence, Poisson regression was fitted to the data, and a negative binomial regression, in case of over dispersion. A step-wise selection procedure was employed to find the most parsimonious model. In the first step, the regression model included maternal religion, a child’s gender, age and nutritional status as explanatory variables. In the second step, only the significant terms in the first step were kept and the evolutions of developmental performance with age was allowed to be curvi-linear (possibly a quadratic association). Furthermore, interactions of the child’s nutritional status with maternal religion, with a child’s gender and age were allowed to examine mediating effects. A significant level of 5% was used. This model building was done for all of the five developmental domains separately. The parsimonious model comprised age as both linear term and quadratic term, nutritional status and their interactions.

Ideally, the difference in developmental performance between malnourished and non-malnourished children was also corrected for maternal education and socio-economic status.Earlier studies have shown association of maternal education with a number of factors such as economic condition [45] and severe malnutrition [46]. But the strong collinearity between these covariates makes the results of a multiple regression model including these factors together untrustworthy. Therefore, for each developmental performance, we opted to investigate three regression models, each focusing on one of these predictors at a time. Model I (as discussed above) studied the relationship between the developmental performance and the nutritional status, Model II between the developmental performance and family socio-economic status, and Model III between the developmental performance and maternal education. In line with the primary objective of this study, more attention was given to model I.

To estimate the delay in developmental performance of SAM children on the different domains of the Denver II-Jimma scale, the number of test items performed by SAM and non-malnourished children at ages three to 70 months were predicted from the regression model. The difference in age of attaining equal number of test items was calculated as an index of developmental delay. A weighted score was calculated by dividing the delay index by the age at which the non-malnourished children perform the same number of items performed by the SAM children. The weighted scores were also converted into percentages, and used for comparisons of different domains at different ages.

An index to quantify social-emotional problems was computed by subtracting the total ASQ:SE scores of the healthy children from that of SAM children at median ages on eight age groups (6, 12, 18, 23.5, 29.5, 37.5, 47.5, 59.5 months). Dividing this problem behavior index by the respective median age resulted in a weighted index. The index was also converted into percentages. The age-specific cutoff was also subtracted from the mean score of SAM child at median age and then divided by the cutoff. This also produced an alternative standard score to determine the deviation of SAM children’s score from the cutoff score. The statistical analysis was performed using STATA Software: Release 12 [47].

## Results

### Characteristics of study participants

A total of 620 (SAM: *n* = 310; non-malnourished: n = 310) children from diverse ethnic, linguistic and religious communities participated in the study (Table 1). Malnourished children (mean age = 30.7 mo and SD = 15.2 mo) are age-matched with non-malnourished children (mean age = 29.6 mo and SD = 15.4 mo). Both SAM and non-malnourished children are equally distributed across eight different age groups. Malnourished children live predominantly in the rural areas, belong to Muslim mothers and represent one dominant ethnic group (the Oromo). Majority (96%) of their mothers have primary or no education, and half (51.6%) of them reported that they belong to low-income family. The non-malnourished group is from Jimma town, and represents diverse religious and ethnic communities with better family income. Nearly half (48%) of their mothers have secondary or higher education, and 58% of them are Christians.

**Table 1:** Demographic characteristics [n(%)] of the participating children (*N* = 620)

	Nutritional status			Nutritional status			Nutritional status	
Characteristics	SAM^a^	Healthy^b^	Characteristics	SAM^a^	Healthy^b^	Characteristics	SAM^a^	Healthy^b^
Sex			Ethnicity			Address		
*Male*	155 (50)	155 (50)	*Amhara*	15 (4.8)	67 (21.6)	*Dedo*	52 (16.8)	–
*Female*	155 (50)	155 (50)	*Dawuro*	3 (0.97)	38 (12.3)	*Jimma town*	44 (14.2)	310 (100)
Age group			*Oromo*	284 (91.6)	132 (42.6)	*Gomma*	16 (5.2)	–
*3–8 mo*	12 (3.9)	14 (4.5)	*Gurage*	1 (0.3)	26 (8.4)	*Mana*	39 (12.6)	–
*9–14 mo*	39 (12.6)	49 (15.8)	*Keficho*	2 (0.6)	14 (4.5)	*Omonada*	20 (6.5)	–
*15–20 mo*	53 (17.1)	52 (16.8)	*Tigre*	0 (0)	8 (2.6)	*Seka*	42 (13.5)	–
*21–26 mo*	38 (12.3)	36 (11.6)	*Wolayita*	1 (0.3)	2 (0.6)	*Serbo*	81 (26.1)	–
*27–32 mo*	30 (9.7)	30 (9.7)	*Yem*	3 (0.97)	0 (0)	*Shabe*	10 (3.2)	–
33–41 *mo*	59 (19)	53 (17.1)	*Other*	1 (0.3)	12 (3.9)	*Others*	6 (1.9)	–
42–53 *mo*	52 (16.8)	52 (16.8)	*Unknown*	0 (0)	11 (3.5)	*SES		
54–65 *mo*	27 (8.7)	24 (7.7)	Maternal religion			*Low or lower*	160 (51.6)	9 (2.9)
Maternal education			*Orthodox*	22 (7.1)	134 (43.2)	*Middle or higher*	149 (48.1)	289 (93.2)
*Illiterate*	244 (78.7)	26 (8.4)	*Protestant*	1 (0.3)	46 (14.8)	*Missing*	1 (0.3)	12 (3.9)
*Primary*	53 (17)	123 (39.7)	*Islam*	287 (92.6)	113 (36.5)			
*Secondary or higher*	12 (3.9)	149 (48)	*Others*	0 (0)	6 (1.9)			
*Unknown*	1 (0.3)	12 (3.9)	*Unknown*	0 (0)	11 (3.5)			

*SAM* ^a^ severely acute malnourished children, *Healthy* ^b^ non-malnourished children, *mo* month, *SES family socio-economic status through self-report by the caregiver

### Predictors of child developmental outcomes

Nutritional status has a significant effect on all five domains of child development. SAM children perform significantly worse than the non-malnourished children on FM, GM, LA, PS, SE (Table 2).

**Table 2:** Three steps^b^ multivariable predictors of performance on five domains of child development

	Covariates					
Developmental Outcomes		^e^Age	Age^2^	^f^Nutri	Nutr#Age	Nutri#Age^2^
^c^Fine motor	^d^IRR	1.036	0.9997	0.7556	1.002	–
	95%CI	[1.032, 1.039]	[0.9996, 0.9998]	[0.7094, 0.8047]	[1.001, 1.004]	–
	*p*-value	0.000	0.000	0.000	0.008	–
^c^Gross motor	^d^IRR	1.042	0.9996	0.7263	–	–
	95%CI	[1.038, 1.046]	[0.9996, 0.9997]	[0.7091, 0.7438]	–	–
	p-value	0.000	0.000	0.000	–	–
^c^Language	^d^IRR	1.050	0.9996^a^	0.9778	0.9829	1.000^a^
	95%CI	[1.046, 1.054]	[0.9996, 0.9997]	[0.8610, 1.110]	[0.9753, 0.9906]	[1.000, 1.000]
	p-value	0.000	0.000	0.730	0.000	0.000
^c^Personal social	^d^IRR	1.053	0.9995	0.7015	1.006	–
	95%CI	[1.049, 1.058]	[0.9994, 0.9996]	[0.6542, 0.7521]	[1.004, 1.007]	–
	p-value	0.000	0.000	0.000	0.000	–
^c^Social-emotional behavior	^d^IRR	1.050	0.9995	3.087	0.9482	1.001^a^
	95%CI	[1.034, 1.067]	[0.9992, 0.9997]	[2.236, 4.261]	[0.9284, 0.9683]	[1.000, 1.001]
	p-value	0.000	0.000	0.000	0.000	0.000

^a^ the estimate lies within the confidence interval when it is in five decimal digits

^b^ Model in Step I analysis comprises age, sex and nutritional status of child, maternal religion; in step II, only significant terms in step I, age as a quadratic term, interactions of nutritional status with maternal religion, with age as a linear term and as a quadratic term were added to step I model; and in step III, non-significant terms in Step II model were dropped beginning from a non-significant interaction between nutritional status and age as a quadratic term

^c^ Multivariable Poisson regression model was fitted; ^d^ Incident rate ratio, analogous to odds ratio, is obtained by exponentiating the coefficient in the Poisson model; ^e^ age of a child (in months); Age^2^, age squared

^f^ ‘Nutri, nutritional status of child’: severely acute malnourished, coded as 1, ‘non-malnourished’ coded as 0 is a reference

**Fig. 2:**
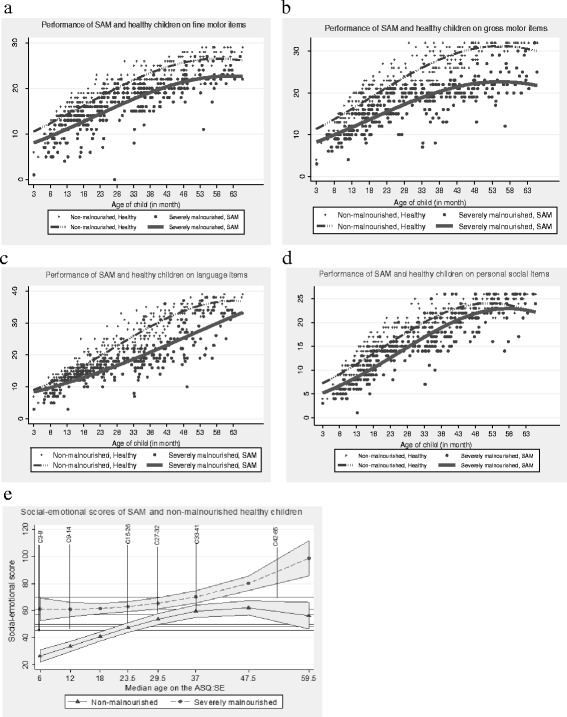
Effects of nutritional status on (**a**)fine motor, (**b**) gross motor, (**c**) language, (**d**) personal social and (**e**) social-emotional development of under-six children

Differences in developmental performance of the severely malnourished and the non-malnourished children are graphically displayed (see Fig. 2). The differences vary depending on the age of the children on all the developmental domains except GM. This is shown by lack of interaction between age and nutritional status for GM (see Table 2) even though the fitted lines within Fig. 2 do not seem to be parallel since the lines are drawn from an exponentiated log scale. The malnourished and the non-malnourished children also differ from each other with respect to maternal religion, but this difference has no significant association with all the developmental outcomes.

**Fig. 3:**
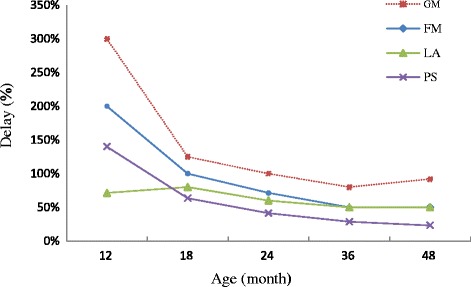
Developmental delay by SAM children on four domains compared to development of non-malnourished children

The most problematic Denver II Jimma domain for SAM children seems to be gross motor. This is observed throughout the whole age range (see Fig. 3). On average, their performance on GM is lowered by 27.4%, 95CI, [−29.1%; −25.6%] compared to the non-malnourished children (Table 2). On the contrary, problems in performances on FM, PS, LA, and SE vary by age as shown by significant interaction between age and nutritional status (Table 2). Very young SAM children show more problems in FM, LA and PS than the older ones (Fig. 3). On the social-emotional domain, they perform worse during early age and at later ages (Fig. 2e and Table 4).

The delay in performance of SAM versus non malnourished children at different ages for GM, FM, LA and PS has been summarized (see Table 3). On fine motor, for instance, a 12 month old SAM child is only capable to perform the skills of a 4 month old healthy child. This is a delay of 8 months or 200%. Very young (until 12 months) SAM children show a delay on GM by 300%, FM by 200%, and PS by 140%. The delay generally decreases with an increase in age except for gross motor. The least affected skill for children under 1 year of age is language (by 71.4%), but after about 18 months of age, it is the personal social skill (Table 3 and Fig. 3).

**Table 3:** Delay in performance of SAM versus non-malnourished children at different ages on motor, language and personal social skills

Age of SAM children	Delay (mo, %)^a^ of SAM children			
	Fine Motor	Gross Motor	Language	Personal Social
12 mo	8 mo (200%)	9 mo (300%)	5 mo (71.4%)	7 mo (140%)
18 mo	9 mo (100%)	10 mo (125%)	8 mo (80%)	7 mo (63.6%)
24 mo	10 mo (71.4%)	12 mo (100%)	9 mo (60%)	7 mo (41.2%)
36 mo	12 mo (50%)	16 mo (80%)	12 mo (50%)	8 mo (28.6%)
48 mo	16 mo (50%)	23 mo (92%)	16 mo (50%)	9 mo (23.1%)

^a^The delay in months (mo) is obtained for each domain by calculating the number of months SAM children lag behind to perform the same number of test items performed by the non-malnourished children. The percentage of delay is calculated by the delay score of the SAM children divided by the age at which non-malnourished children perform the same number of items multiplied by hundred

For language, the delay increases slightly before the age of 2 years and then decreases (Fig. 3). From age three onwards, it seems that both fine motor and language are equally affected. Generally, the degree of delay on the four domains decrease with an increase in age.

The predicted count scores of SAM children on social-emotional (SE) domain are systematically above the age specific cutoff scores and the scores of the non-malnourished children (Table 4 **,** Fig. 2e). Scores above the age specific cut offs indicate the presence of social–emotional problems. This effect is more severe for SAM children in the first age category (3–8 mo) and those in the last category (54–65 mo) (Fig. 2e).

**Table 4:** Social-emotional performance of SAM versus non-malnourished children at median ages on ASQ:SE

	^a^Median age (in month)							
	6	12	18	23.5	29.5	37.5	47.5	59.5
SAM children’s ASQ:SE score	61.1	60.8	61.5	62.9	65.4	70.1	80.1	98.6
^b^ASQ:SE cutoff score	45	48	50	50	57	59	70	70
^c^ Deviation from cutoff (%)	16.1 (35.8%)	12.8 (26.7%)	11.5 (23%)	12.9 (25.8%)	8.4 (14.7%)	11.1 (18.6%)	10.1 (14.4%)	28.6 (40.9%)
^d^Difference: SAM versus healthy	34.5 (575%)	27.3 (227.5%)	20.6 (114.4%)	15.5 (66%)	11.6 (39.3%)	10.5 (28%)	18.1 (38.1%)	42.3 (71.1%)

*ASQ:SE* ages and stages questionnaires: social-emotional, *SAM* severely acutely malnourished

^a^ The median of the age ranges on the eight age groups on ASQ:SE; ^b^ Child’s ASQ:SE score greater or equal to a cutoff score marks presence of problem behavior; ^c^ Deviation score is a distance of SAM child’s score from the cutoff score. Deviation percentage is calculated by dividing it by the cutoff score and then multiplying by hundred; ^d^ ‘Difference’ is calculated by referring to the healthy children (subtracting the social-emotional score of the non-malnourished children from that of SAM children); dividing the ‘difference’ by the median age at comparison and multiplying it by hundred gives its percentage

In further analyses, the effects of maternal education and family socio-economic status on the developmental outcomes were examined separately using multivariable models comprising child’s gender and age and their interactions as covariates (see Additional file 1: Table S1). The result showed a lack of a significant relationship between a child’s gender and child developmental outcomes, but maternal education and family socio-economic status were found to be significant predictors in the five domains of child development.

## Discussion

Though the first 3 years are generally considered very critical, some years later are also very much crucial for children’s holistic development. Yet most recent studies on developmental effects of severe acute malnutrition focused mainly on young children not older than 24 months of age. No study has concurrently addressed and compared various developmental dimensions among SAM children of different ages, particularly during its acute stage. Consequently, much is not known about the degree to which the different dimensions of child development is impaired during the acute stage of SAM in children under 6 years of age.

The present study examined the effect of SAM on five domains of development in children 3 months to 6 years of age. Severely acutely malnourished children performed worse on personal-social, fine motor, language, gross motor skills and social-emotional competences compared to age and sex-matched non-malnourished healthy children. More specifically the study revealed that 1) motor skills are the most but personal social are the least affected domains assessed on the Denver II-Jimma, and, 2) there is a differential effect of age on all the domains except on gross motor development of SAM children under 6 years of age.

Earlier studies compared the development of hospitalized SAM children with protein energy malnutrition with hospitalized non-malnourished control children of other illnesses. The malnourished children were markedly behind the controls [23]. A review of earlier studies also indicated that all developmental levels are extremely low in the acute stage and generally improve during recovery [48]. Our study is consistent with earlier studies which showed that SAM negatively affects motor skills [18, 19]. Earlier studies, however, did not specify the areas of development worst or least affected. The present study reveals that the motor skills in general, and the gross motor in particular, are more seriously affected than the language and the personal social skills of SAM children. In fact, more severe effects on motor skills (gross) is expected since severe acute malnutrition reduces muscle mass. Muscle atrophy could reduce a child’s physical activity and deter further explorations and interactions with environment. This might in turn hamper not only the development in the gross motor but also in the fine motor, the personal social and the language skills.

It is now known that the effect of SAM at early life of a child is sustained to later ages. Children with histories of either marasmus or kwashiorkor during their first year of life [15, 17, 18] scored at later ages significantly lower on national high school examination [15], intellectual performance [17–19] than healthy children. Adults who had experienced an episode of moderate to severe protein-energy malnutrition during the first year of life scored significantly lower than the healthy controls on measures of cognitive flexibility and concept formation, as well as initiation, verbal fluency, working memory, processing speed, and visuospatial integration [49].

Our study shows that the degree of developmental lag in SAM children during the acute stage varies depending on the child’s age. Age related effects of SAM on child development have been documented in some retrospective studies. For instance, there was a lack of significant difference on psychomotor performance between a group of children aged from 6 to 12, who suffered from kwashiorkor during infancy and control groups having no infantile malnutrition [20]. Similarly, a study compared children admitted to hospital with undernutrition during the first year of life. Three to four years later, the mean developmental quotient of children treated in the first 4 months of life, and the control ones was similar. However, there was a difference in the developmental quotient of the control and those treated for undernutrition after 4 months of age [50]. Another study compared subjects of 2 to 21 years of age, who had been severely malnourished and hospitalized during the first 6 months with control siblings on intellectual performance, sensory motor abilities and social adaptation. No significant difference was found for the older subjects. It was argued that the significant effect of infantile malnutrition prevails only in children aged between 2 to 5 years following their episode of malnutrition, and that there is no significant difference after the age of 5 years [21].

Our study also shows that hospitalized SAM children have more social-emotional problems than the non-malnourished healthy children. Such behavior problems during an acute episode may persist to later life. That has been shown in school age children with histories of malnutrition during early childhood. They showed greater behavioral problems than matched controls and, to a lesser extent, than siblings [48]. Similarly, elevated conduct problems [51] and depressive symptoms [52] were reported in youth with histories of protein-energy malnutrition during the first year of life.

Interpreting our results, however, has to take the following drawbacks into account. Majority of the SAM children came from illiterate, low income and Muslim families living in rural areas and were assessed while they were in hospital. On the other hand, the non-malnourished children come from family with better socioeconomic status and mostly literate mothers of various religious backgrounds. They live in Jimma town and were assessed mostly at home setting. As this study did not control for the effect of hospitalization, the result may not show the developmental profiles of SAM children in non-hospital settings. Nonetheless, the study shows the extent to which SAM children of different ages admitted to hospital for treatment are delayed in different dimensions of development compared to non-malnourished healthy children.

## Conclusions

The study reveals that the developmental performance of SAM children is seriously affected during the acute stage. This effect is multidimensional and age-dependent. Hence rehabilitation of SAM children should be multi-dimensional, age-specific and focus on strengthening of motor skills during early age. Interventions at health institution have to transcend the mere goal of achieving growth and survival as prime measures of successful health outcomes and include development as an important component as well. Future research has to examine the effect of intervention on different dimensions of development among children of different ages.

## Additional file

Additional file 1: Multivariable predictors of developmental performance on four domains of child developmenta. (DOCX 38 kb)

## Abbreviations

ASQ:SE: Ages and stages questionnaires: social-emotional
FM: Gross motor
GM: Gross motor
LA: Language
MUAC: Mid-upper circumference
MUACZ: Mid-upper arm circumference z score
PS: Personal social
SAM: Severe acute malnutrition
SE: Social-emotional
WAZ: Weight-for-age z score
