# Supplementary material for: Developmental performance of hospitalized severely acutely malnourished under-six children in low- income setting
Source: BMC Pediatr. 2017 Nov 28;17:197. doi: 10.1186/s12887-017-0950-5 (PMC5704634; doi:10.1186/s12887-017-0950-5)
Supplement: Additional file 1: — Multivariable predictors of developmental performance on four domains of child developmenta. (DOCX 38 kb) [file 12887_2017_950_MOESM1_ESM.docx]

| **STable 1** Multivariable predictors of developmental performance on four domains of child development**^a^** | | | | | | | | | | | | | | | | |
| --- | --- | --- | --- | --- | --- | --- | --- | --- | --- | --- | --- | --- | --- | --- | --- | --- |
| **predictors** | **Model** | **Fine motor** | | | **Gross motor** | | | **Language** | | | **Personal social** | | | **Social-emotional** | | |
|  |  | IRR**^b^** | 95%CI | p-value | IRR**^b^** | 95%CI | p-value | IRR**^b^** | 95%CI | p-value | IRR**^b^** | 95%CI | p-value | IRR**^b^** | 95%CI | p-value |
| Sex^c^ |  |  |  |  |  |  |  |  |  |  |  |  |  |  |  |  |
| Female | I | [1.020] | [0.9868, 1.042] | 0.093 | 0.9953 | [0.9721 , 1.019] | 0.693 | 1.012 | [0.9867, 1.038] | 0.352 | 1.022 | [0.9932, 1.051] | 0.136 | 0.9877 | [0.8769, 1.112] | 0.838 |
| Age* | I | 1.036 | [1.032, 1.040] | 0.000 | 1.0412 | [1.037, 1.046] | 0.000 | 1.042 | [1.039, 1.046] | 0.000 | 1.053 | [1.049, 1.058] | 0.000 | 1.010 | [0.9987, 1.022] | 0.083 |
| Age#Age | I | 0.9997 | [0.9996, 0.9998] | 0.000 | 0.9996^¥^ | [0.9996, 0.9997] | 0.000 | 0.9997¥ | [0.9997, 0.9998] | 0.000 | 0.9995 | [0.9994, 0.9996] | 0.000 | 0.9999 | [0.9998, 1.000] | 0.348 |
| Nutritional Status**^d^** |  |  |  |  |  |  |  |  |  |  |  |  |  |  |  |  |
| SAM** | I | 0.7600 | [0.7083, 0.8155] | 0.000 | 0.7148 | [0.6692, 0.7633] | 0.000 | 0.7620 | [0.7128, 0.8146] | 0.000 | 0.6990 | [0.6492, 0.7527] | 0.000 | 1.724 | [1.415, 2.100] | 0.000 |
| SAM # Female | I | 0.9880 | [0.9445, 1.034] | 0.601 | 1.020 | [0.9720, 1.069] | 0.427 | 0.9967 | [0.9449, 1.051] | 0.905 | 1.004 | [0.9555, 1.056] | 0.865 | 0.9185 | [0.7892, 1.070] | 0.272 |
| SAM#Age | I | 1.002 | [1.001, 1.004] | 0.008 | 1.000 | [0.9986, 1.002] | 0.822 | 1.000 | [0.9985, 1.002] | 0.921 | 1.006 | [1.004, 1.007] | 0.000 | 0.9952 | [0.9901, 1.000] | 0.065 |
| Sex**^c^** |  |  |  |  |  |  |  |  |  |  |  |  |  |  |  |  |
| Female | II | 1.014 | [0.9843, 1.045] | 0.353 | 1.000 | [0.9648, 1.037] | 0.988 | 1.024 | [0.9871, 1.061] | 0.208 | 1.018 | [0.9867, 1.050] | 0.265 | 0.9103 | [0.8237, 1.006] | 0.065 |
| Age | II | 1.035 | [1.031, 1.039] | 0.000 | 1.040 | [1.035, 1.045] | 0.000 | 1.041 | [1.036, 1.046] | 0.000 | 1.054 | [1.049, 1.058] | 0.000 | 1.010 | [0.9976, 1.022] | 0.115 |
| Age#Age | II | 0.9997^¥^ | [0.9997, 0.9998] | 0.000 | 0.9997^¥^ | [0.9995, 0.9997] | 0.000 | 0.9997^¥^ | [0.9997, 0.9998] | 0.000 | 0.9995^¥^ | [0.9995, 0.9996] | 0.000 | 0.9999 | [0.9997, 1.000] | 0.235 |
| SES**^e^** |  |  |  |  |  |  |  |  |  |  |  |  |  |  |  |  |
| Low *** | II | 0.7832 | [0.7110, 0.8626] | 0.000 | 0.7447 | [0.6763, 0.8200] | 0.000 | 0.8294 | [0.7588, 0.9065] | 0.000 | .7154 | [0.6531, 0.7835] | 0.000 | 1.332 | [1.082, 1.639] | 0.007 |
| Low# Female | II | 1.004 | [0.9492 1.063] | 0.877 | 1.035 | [0.9658, 1.109] | 0.329 | 0.9698 | [0.9020, 1.043] | 0.407 | 1.032 | [0.9735, 1.095] | 0.287 | 1.072 | [0.9197, 1.251] | 0.372 |
| Low# Age | II | 1.003 | [1.001,  1.005] | 0.007 | 1.002 | [0.9992, 1.004] | 0.189 | 1.000 | [0.9983, 1.003] | 0.692 | 1.005 | [1.003, 1.008] | 0.000 | 0.9985 | [0.9931, 1.004] | 0.581 |
| Sex**^c^** |  |  |  |  |  |  |  |  |  |  |  |  |  |  |  |  |
| Female | III | 1.019 | [0.9901, 1.049] | 0.198 | 0.9867 | [0.9543, 1.020] | 0.431 | 1.003 | [0.9710, 1.036] | 0.868 | 1.023 | [0.9924, 1.054] | 0.144 | 0.8978 | [0.8005, 1.007] | 0.065 |
| Age | III | 1.037 | [1.033 1.041] | 0.000 | 1.044 | [1.039, 1.049] | 0.000 | 1.044 | [1.040, 1.049] | 0.000 | 1.055 | 1.050, 1.060 | 0.000 | 1.007 | [0.9952, 1.019] | 0.245 |
| Age#Age | III | 0.9997^¥^ | [0.9996,  0.9997] | 0.000 | 0.9996^¥^ | [0.9996, 0.9997] | 0.000 | 0.9997 | [0.9996, 0.9998] | 0.000 | 0.9995 | [0.9994, 0.9996] | 0.000 | 0.9999 | [0.9998, 1.000] | 0.557 |
| Matenal Education**^f^** |  |  |  |  |  |  |  |  |  |  |  |  |  |  |  |  |
| Illiterate | III | 0.8290 | [0.7648, 0.8986] | 0.000 | 0.8052 | [0.7420, 0.8738] | 0.000 | 0.8031 | [0.7465, 0.8640] | 0.000 | 0.7410 | [0.6845, 0.8021] | 0.000 | 1.443 | [1.177, 1.769] | 0.000 |
| Illiterates # female | III | 0.9801 | [0.9321, 1.031] | 0.433 | 1.037 | [0.9797, 1.098] | 0.209 | 1.019 | [0.9592, 1.083] | 0.537 | 0.9947 | [0.9434, 1.049] | 0.845 | 1.093 | [0.9385, 1.272] | 0.253 |
| Illiterate # Age | III | 1.001 | [0.9991, 1.003] | 0.292 | 0.9984 | [0.9964, 1.000] | 0.097 | 0.9995 | [0.9977, 1.001] | 0.596 | 1.004 | [1.003, 1.006] | 0.000 | 0.9970 | [0.9917, 1.002] | 0.273 |
| Age*****, age of a child (in months); SAM**, severe acute malnourished; SES, socio-economic status; Low***, very low/ low SES versus Middle/ High SES,  ^¥^ lies within the confidence interval range with five decimal digits.  **^a^** Multivariable Poisson regression model was fitted; **^b^**IRR, Incident rate ratio, is analogous to odds ratio and is obtained by exponentiating the coefficient in the Poisson model.  Model I:covariates are nutritional status, sex, age, age#age, interactions of nutritional status with age and with child’s sex**;**  Model II: covariates in model II are socio-economic status, sex, age, age#age, interactions of socio-economic status with age and with child’s sex;  Model III: covariates in model III are educational status, sex, age, age#age, interactions of educational status with age and with child’s sex;  **^c^** ‘male’ is a reference; **^d^** ‘non-malnourished’ is a reference; **^e^** ‘middle / high’ is a reference; **^f^** ‘Primary or above primary’ is a reference; | | | | | | | | | | | | | | | | |
